# *Nuts'a'maat shqwaluwun* — Knitting ways of life with Indigenous research principles to examine preterm birth in Quw'utsun

**DOI:** 10.17269/s41997-025-01028-2

**Published:** 2025-04-16

**Authors:** Tˈultunaat Lydia Seymour, Doreen Peter, Eugenia Tinoco, Maia Thomas, Lynsey Johnny, Brenda Yuen, Liz Spry, Fairlie Mendoza, Marnie Elliott, Barbara Webster, Shannon Waters, Diane Sawchuck, Ashley Simpson, Jennifer Leason, Patricia A. Janssen, Jennifer B. Murray

**Affiliations:** 1 Tsˈewulhtun Health Centre, Cowichan Tribes, Duncan, BC Canada; 2 First Nations Health Authority, Vancouver, BC Canada; 3 Office of the Provincial Health Officer, Government of British Columbia, Victoria, BC Canada; 4 Island Health, Victoria, BC Canada; 5 Department of Political Science, University of Calgary, Calgary, AB Canada; 6 School of Population and Public Health, Faculty of Medicine, University of British Columbia, Vancouver, BC Canada; 7 BC Children’s Hospital Research Institute, Vancouver, BC Canada

**Keywords:** Indigenous Peoples, Community-based participatory research, Indigenous Peoples’ rights, Canada, Preterm birth, Peuples autochtones, Recherche participative basée sur la communauté, Droits des peuples autochtones, Canada, Naissance prématurée

## Abstract

**Setting:**

The Quw'utsun Preterm Birth Study used a community-led and participatory action research methodology to investigate preterm birth in Quw'utsun, a First Nations community in Cowichan Valley, British Columbia (BC). Quw'utsun people and staff from the community’s Ts'ewulhtun Health Centre partnered with the BC First Nations Health Authority, Island Health (regional health authority), and the University of British Columbia to develop *Nuts'a'maat shqwaluwun* (one heart, one mind), a framework for conducting research activities.

**Intervention:**

Guided by Elders, *Nuts'a'maat shqwaluwun* incorporated Quw'utsun standards for research ethics by knitting together *snuw'uy'ulh* (ways of life), such as *Stsi'elh stuhw tu Sul-hween* (honour the Elders), with federal policy for ethical conduct of research involving Indigenous people. Situating the study at Cowichan Tribes strengthened the community’s authority to lead.

**Outcome:**

The framework, *Nuts'a'maat shqwaluwun*, fostered a research environment where we could *Ti'tul'atul' tst* (learn from one another). We learned to bring our knowledges together to conduct the study in ways that respected snuw'uy'ulh. This research was meaningful to Quw'utsun people because snuw'uy'ulh were respected. Our partnerships resulted in the first-ever report of preterm birth rates and risk factors among Quw'utsun people. Knowledge translation activities enhanced community access to results.

**Implications:**

Indigenous Peoples have an inherent and legislated right to self-determination, including the right to lead research involving them. Several principles within *Nuts'a'maat shqwaluwun* enabled Quw'utsun people to lead this research: (1) trusting relationships; (2) respecting community-specific ways of life; (3) community ownership and access to data; and (4) training opportunities to lead research.

## Résumé

**Lieu:**

L’étude *Quw'utsun Preterm Birth Study* a fait appel à une méthode de recherche-action communautaire pour enquêter sur les naissances avant terme chez les peuples Quw'utsun, une communauté des Premières nations de la vallée du même nom en Colombie-Britannique. Des personnes Quw'utsun et des membres du personnel du Ts'ewulhtun Health Centre se sont associés à la BC First Nations Health Authority (régie de la santé des Premières Nations de la province), à Island Health (régie régionale de la santé) et à l’Université de la Colombie-Britannique pour élaborer un cadre pour les activités de recherche intitulé *Nuts'a'maat shqwaluwun* (un cœur, un esprit).

**Intervention:**

Guidé par des aînés, le cadre *Nuts'a'maat shqwaluwun* a intégré les normes des peuples Quw'utsun dans l’éthique de la recherche en entrelaçant leurs principes de vie (*snuw'uy'ulh*), comme le respect envers les aînés (*Stsi'elh stuhw tu Sul-hween*), dans la politique fédérale d’éthique de la recherche impliquant des peuples autochtones. Le fait d’avoir situé l’étude dans les tribus Cowichan a renforcé l’autorité de la communauté sur la conduite de l’étude.

**Résultat:**

Le cadre *Nuts'a'maat shqwaluwun* a favorisé un milieu de recherche où il était possible d’apprendre les uns des autres (*Ti'tul'atul' tst*). Nous avons appris à réunir nos savoirs pour mener l’étude en respectant les *snuw'uy'ulh*. Cette recherche était significative pour les peuples Quw'utsun, car les *snuw'uy'ulh* ont été respectés. Nos partenariats ont donné lieu au tout premier rapport sur les taux de naissance avant terme et leurs facteurs de risque chez les peuples Quw'utsun. Des activités d’application des connaissances ont amélioré l’accès de la communauté aux résultats de l’étude.

**Conséquences:**

Les peuples autochtones ont un droit inhérent et légiféré à l’autodétermination, dont le droit de diriger la recherche qui les concerne. Plusieurs principes du cadre *Nuts'a'maat shqwaluwun* ont permis aux peuples Quw'utsun de diriger l’étude: 1) des liens de confiance; 2) le respect des principes de vie de la communauté; 3) l’appartenance des données à la communauté et son accès aux données; et 4) des possibilités de formation sur la direction de l’étude.

## Setting

Indigenous Peoples have an inherent right to self-determination, recognized in federal law (Government of Canada, 2021) and provincial law in British Columbia (BC) (Province of British Columbia, 2019), and affirmed internationally (United Nations Declaration on the Rights of Indigenous Peoples (United Nations, 2007)). Conflicting with the right to self-determination, research around Indigenous people’s health has predominantly used Western disease-based models and research methodologies (Kovach, 2021), and has excluded or minimally involved Indigenous Peoples in the conduct of research (Schnarch, 2004). Resulting evidence has lacked contextualization of how colonization and colonialism influenced the health of Indigenous Peoples and minimized Indigenous knowledges and ways of knowing (epistemologies). This evidence has often perpetuated false, discriminatory, or racist narratives (Ermine et al., 2004). In extreme cases, medical experimentation occurred without informed consent (Mosby, 2013).

In response, policies, principles, and frameworks (“principles”) for conducting ethical research involving Indigenous Peoples have emerged. In Canada, the Tri-Council Policy Statement 2 (TCPS-2) established requirements for ethical conduct of research involving Indigenous Peoples (Government of Canada, 2022). This includes a requirement to engage with the relevant Indigenous community; respect the distinct community customs and codes of practice within First Nations, Inuit, and Métis communities; respect governing authorities; ensure mutual benefit in research; and recognize the role of Elders and other cultural advisors. A commonly used code of practice in First Nations communities is Ownership, Control, Access, and Possession (OCAP®), developed for the First Nations Regional Health Survey (Schnarch, 2004). OCAP® specifies that First Nations require ownership, control, access, and possession of research, including data, that involves them.

**Fig. 1:**
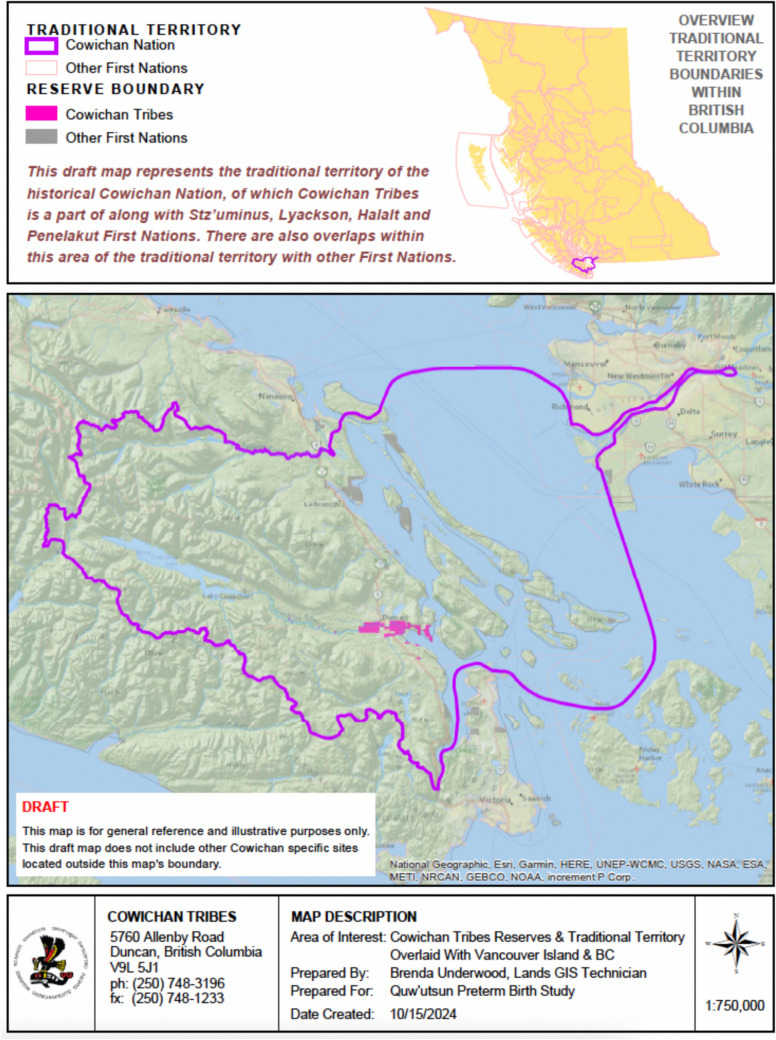
Map of traditional territory of the Cowichan Nation

*Quw'utsun mustimuhw* (Cowichan people) are *Hwuhwilmuhw* (First Nations people) from seven villages—*Kwa'mutsun*,* Qw'umi'yiqun'*,* Xwulqw'selu*,* S-amunu*,* Lhumlhumuluts'*,* Xinupsum*,* Tl'ulpalus*—that collectively include over 5300 community members known as Cowichan Tribes. Quw'utsun mustimuhw have lived on their territory, Squw'utsun tumuhw (land of Cowichan people), for thousands of years (Fig. 1). This territory is also known as southern Vancouver Island, Cowichan Valley, British Columbia (BC), Canada. The traditional language is *Hul'q'umi'num'* and the *snuw'uy'ulh* are the Quw'utsun and family-specific ways of life (Table 1) (Health and Welfare Canada, 1986). European settlers landed in Squw'utsun tumuhw in 1862 in search of gold, timber, and farmland (Marshall, 1999). Colonial legislation included land theft and the Indian Residential School system that forcibly removed Indigenous children from their families to assimilate them into white colonial society (Government of Canada, 1996, 2015). Examples of ongoing colonialism include the removal of Indigenous children from their families at birth and in childhood (National Inquiry MMIWG, 2020). Colonialism and colonial legislations continue to have devastating consequences on the lives of Quw'utsun mustimuhw and other Indigenous Peoples. We use *Hul'q'umi'num'* words to convey that knowledge in Quw'utsun is shared in the traditional language, to support language revitalization, and to remind readers that forced use of English or French is a weapon of colonialism in Indigenous communities (Government of Canada, 1996) (Table 2).

**Table 1:** Quw'utsun Snuw'uy'ulh (ways of life) and English translation

Hul'q'umi'num' language	English language
*Stsi'elh stuhw tu Sul-hween*	Honour the Elders
*Mukw' tu shhwa'luqwa 'o' tth'ele's tu shhwuli*	Family is the heart of life
*Tl'i' to' mukw' mustimuhw*	Each person is important
*To'mukw' ni' 'u tun'a tumuhw 'i' ni' ts'lhnuts'atul – mukw' tst 'o' hiiya'yutul*	Everything in nature is part of our family—we are all relatives
*To'mukw' 'i' 'u tun'u tumuhw 'I' 'o' la'lum'utul tst*	Live in harmony with nature
*Hwial'asmut tun'a tumuhw*	Take care of the Earth and take only what you need
*Hwialasmut ch tun' s-ye'lh*	Take care of your health
*Yath ch 'o' lhq'il'*	Be positive
*'lyusstuhw tun'a kweyul*	Enjoy today
*Thuluqtul ch 'u kwthun' kwunmun*	Share what you have
*Lhq'il ch 'u kwthun' suluthut 'i' tun' sqwal*	Be honest and truthful in all you do and say
** − **	Do the best you can do, be the best you can be
*Ti'tul'atul' tst*	Learn from one another
*Thuthi'stuhw tun' shqwaluwun 'u tun' ts'lhmustimuhw*	Be true with your feelings and to another
*'I' yet ch kwthun' syuw'i'na'qw 'i' kwthu ni' xtsutus*	Respect your leaders and their decisions
*'I' yet ch kwthun' sht'unuxun*	Respect your neighbours
*Ha ch ts-tamut 'i' nuwus ch 'o' tl'ul'im't*	Take responsibility for your actions
*Ts'its'uw' atul. Nuts'umat kwun' syaays sh-hwuys kwtho' mukw'*	Help one another. Work together for the good of all
*Hwial'asmut tu tumuhw*	Take care of the Earth — all things are connected
*Ts'iiyulh ch 'u tu ni' s-aamustham-mut*	Give thanks for what you have been given

Quw'utsun Elders Philomena Williams and Laureen Charlie reviewed final translations, adapted from *Quw'utsun Hul'q'umi'num' Category Dictionary*. Copyright © 2007, Cowichan Tribes. The Hul'q'umi'num' writing is still being translated and indicated here with a dash

**Table 2:** Hul'q'umi'num' language and English translation

Hul'q'umi'num' language	English language
Hwialusmutul'	Looking after one another
Hwulmuhw	First Nations person
Hwuhwilmuhw	First Nations people
Mulen'	Fathers
Mustimuhw	People
Naan 'o' tl'i' s-yaays	This work is meaningful
Nults'almuhw	People who are First Nations from another community or non-Indigenous
Nuts'a'maat shqwaluwun	One heart, one mind
Quw'utsun	Cowichan
Slhunlheni'	Women
Snuw'uy'ulh	Ways of life (in Cowichan)
Squw'utsun tumuhw	Land of Cowichan people
Sul-hween	Elder or Elders
Tuletun'	Mothers
Ts'ewulhtun	To help one another (in reference to Ts'ewulhtun Health Centre)
Xpey'	Cedar

Quw'utsun Elders Philomena Williams and Laureen Charlie reviewed final translations, adapted from *Quw'utsun Hul'q'umi'num' Category Dictionary*. Copyright © 2007, Cowichan Tribes

Worldwide and in Canada, Indigenous women are at increased risk of preterm birth (Smylie et al., 2010), defined as birth before 37 weeks of gestation (Ohuma et al., 2023). Preterm birth increases risk of neonatal mortality and life-long morbidity. Differences in perinatal health outcomes between Indigenous and non-Indigenous people have been linked to the intergenerational impacts of colonialism (Leason & Sutherland, 2022).

In 2018, staff at Cowichan Tribes’ Ts'ewulhtun Health Centre were concerned about preterm birth and conducted a manual review of health records of community members. They found that the rate of preterm birth was up to three times higher than for other First Nations Peoples in Canada (Sheppard et al., 2017). The reason for these elevated rates of preterm birth is not known. In response, the Quw'utsun Preterm Birth Study was initiated to “bring healthy birth back to Quw'utsun”. Building on our ethical framework to “bring ethics review home to* Cowichan*” (Cowichan Tribes, 2021), we describe our research framework, *Nuts'a'maat shqwaluwun*, that was guided by Sul-hween (Elders) to conduct the study. Results of data analyses will be shared in future publications.

### Authorship

T'ultunaat (Lydia Seymour) (LS)^1^ and Doreen Peter (DP), who are both Sul-hween, led this study. Both served their community for 30 years as community health workers and LS was a licensed practical nurse. LS and DP are proudly great-grandmothers, grandmothers, mothers, and aunties to many. LS and DP’s authorship positions reflect the study team’s way of honouring the Elders’ guidance of this study. As the eldest, LS is the first author, reflecting that the Eldest Sul-hween are honoured in Quw'utsun.

The co-authors are members of the Quw'utsun Preterm Birth Study (“Study Team”) and include (1) *Hwuhwilmuhw* who are Quw'utsun mustimuhw (LS, DP, ET, MT, LJ, SW, and ME); and (2) *Hwuhwilmuhw* who are not Quw'utsun community members (AS and JL), and people who are not *Hwuhwilmuhw* (JM, BY, LSS, FM, BW, DS, and PJ). “Our” refers to the perspective of the study team as a whole and “we” refers to the perspective of study team members who are Nults'almuhw (people who are First Nations from another community or non-Indigenous). The primary writer was JM, who is a fourth-generation settler of Western European ancestry. This paper is a component of her doctoral degree at University of British Columbia’s (UBC) School of Population and Public Health.

## Building the framework: *Nuts'a'maat shqwaluwun*

To initiate the Quw'utsun Preterm Birth Study, staff at Ts'ewulhtun Health Centre partnered with (1) the First Nations Health Authority (FNHA), a health authority that delivers health programs to, and partners with, First Nations in BC; and (2) Island Health, the regional health authority for Vancouver Island. The study is community-led and uses a participatory action research methodology. The study took place at Ts'ewulhtun Health Centre as a part of the Hwialusmutul' (looking after one another) team that supports healthy lifestyles for Quw'utsun mustimuhw.

Cowichan Tribes’ Chief and Council approved this study with a Band Council Resolution following recommendation for approval from Ts'ewulhtun Health Centre’s Health Advisory Committee. The Health Advisory Committee recommended that LS and DP lead the study, as each had served their community for decades and were respected as cultural advisors. LS and DP established a Quw'utsun-led procedure for ethical review to increase the community’s control of the conduct of the study (UBC REB: H19-023530) (Cowichan Tribes, 2021). A collaborative, in-person, and on-territory research ethics procedure “started the study on the right path” (DP) with community members as witnesses. The study team, ethics board representatives, and health system partners were honoured in a blanketing ceremony to recognize each person’s commitment to the study.

**Fig. 2:**
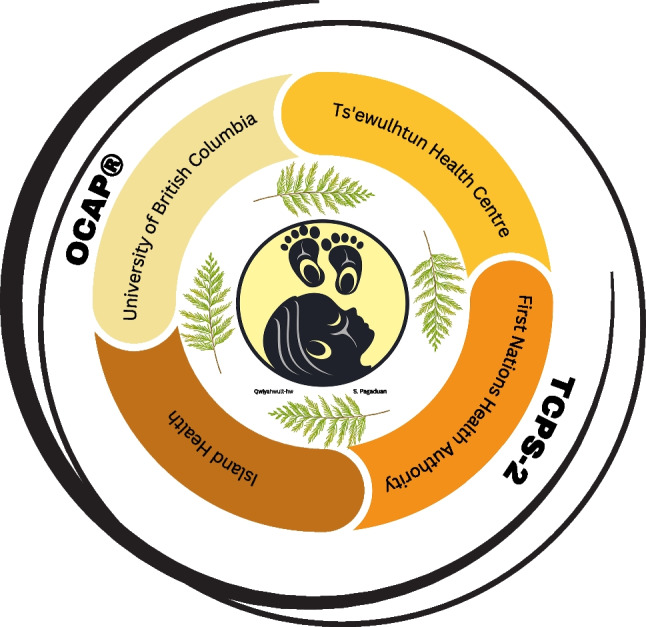
Framework for the Quw'utsun Preterm Birth Study: Nuts'a'maat shqwaluwun. Description: This figure shows how we are creating the work together as nuts'a'maat shqwaluwun. At the centre is the study logo, representing the overall goal of the Quw'utsun Preterm Birth Study to “Bring healthy birth back to Quw'utsun”. Four cedar branches around the logo represent how the Sul-hween (Elders) shared snuw’uy’ulh (ways of life) to guide the study. There are four branches to depict how four directions are important to Quw'utsun people. Each of the partners (Ts'ewulhtun Health Centre, First Nations Health Authority, Island Health, and University of British Columbia) supports the goals of the study and they are in a circle to depict our work together. The words OCAP® (Ownership, Control, Access and Possession) and TCPS-2 (Tri-Council Policy Statement – Chapter 2) represent principles of Indigenous health research that were knit into this framework. The outer circle is unfinished to show how this framework is in progress and will evolve and change in the future

LS and DP then guided us to create a broader research framework to conduct study activities. Our framework of working together as *nuts'a'maat shqwaluwun* (one heart, one mind) meant that we incorporated Quw'utsun standards for research ethics and knit together the snuw'uy'ulh with principles for conducting Indigenous health research, especially TCPS-2 and OCAP® (Government of Canada, 2022; Schnarch, 2004) (Fig. 2). There are family-specific snuw'uy'ulh in Quw'utsun. Some families teach to avoid winding wool in pregnancy while other families teach to avoid knitting altogether in pregnancy. Our use of the word “knit” acknowledges the Quw'utsun way of creating the Cowichan Sweater, which combined traditional weaving with European knitting techniques (Stopp, 2012).

### Study team

**Fig. 3:**
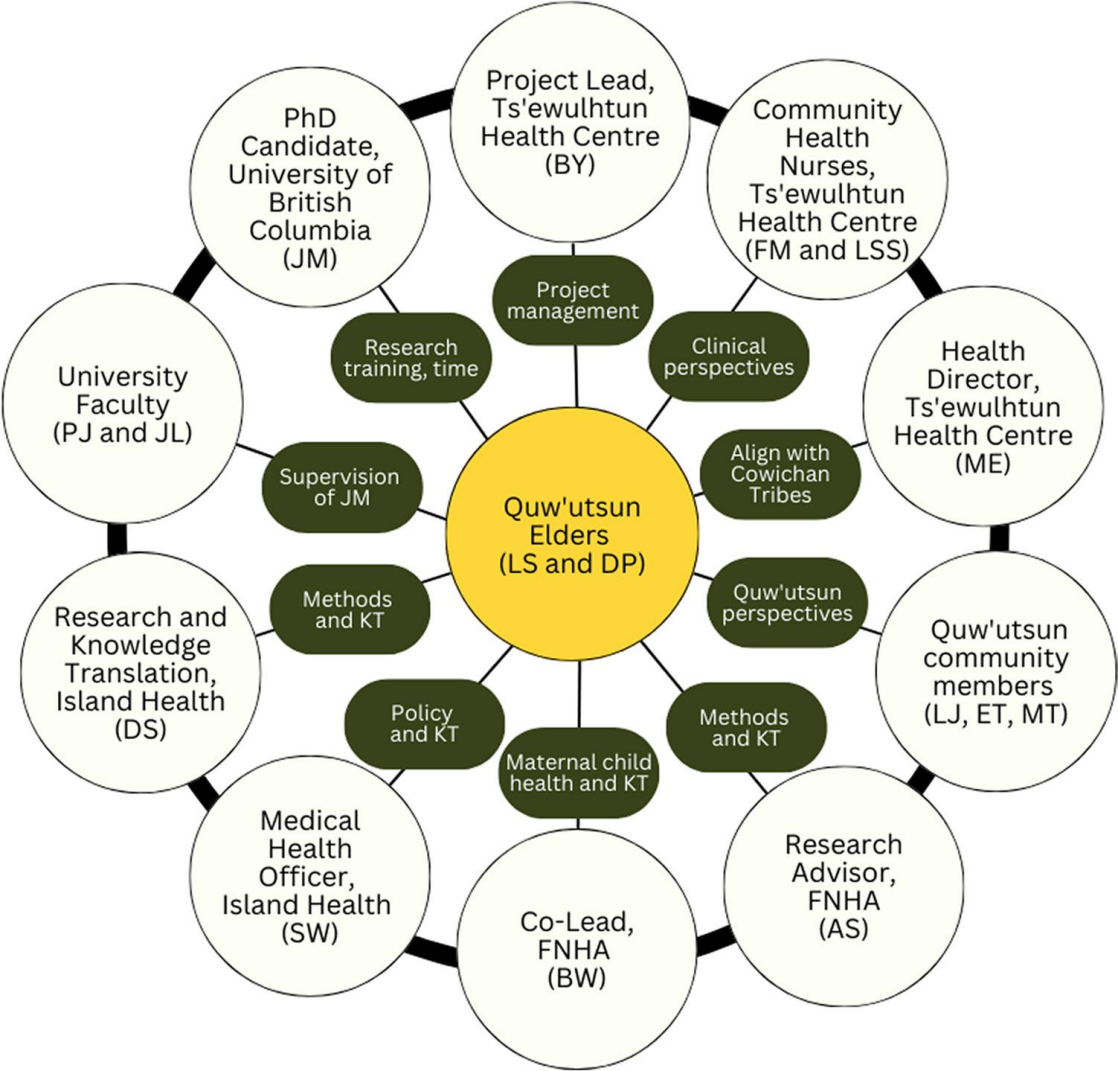
Study team for the Quw'utsun Preterm Birth Study. Abbreviation: FNHA, First Nations Health Authority; KT, knowledge translation

Our roles and how we supported LS and DP to guide this study are depicted in Fig. 3. The Project Lead (BY), employed at Ts'ewulhtun Health Centre, led project management. The Co-Lead (BW), employed at FNHA, brought expertise as clinical nurse specialist for maternal and early child health and allocated in-kind support that included integration of knowledge translation with ongoing work on maternal child health and wellness at FNHA. As a PhD candidate, JM contributed her research training and time. JM created a memorandum of understanding with Cowichan Tribes outlining her role as a community-based scholar and commitment to respecting Quw'utsun ethical standards and OCAP®. To strengthen her relationships with the study team, JM resided in the Cowichan Valley for several years.

A Quw'utsun Research Advisory Committee met monthly to advise on study activities. Three Quw'utsun community members were selected by LS and DP for their previous leadership activities. The Ts'ewulhtun Health Centre’s Director (ME) reviewed all results and integrated the study with activities of the health centre. Two community health nurses, FM and LSS, brought decades of experience working for Cowichan Tribes and provided community-specific context on creating data collection tools and preparation of results. Four health system partners allocated in-kind support. From Island Health, SW (former Medical Health Officer, Cowichan Valley Region) and DS (Lead for Evidence, Evaluation and Knowledge Translation) contributed to planning integration of findings into the health authority’s priorities and expertise on research methods. SW is a member of Stz'uminus First Nation with family ties to Quw'utsun. From FNHA, AS (Research Advisor) supported the Co-Lead (BW). PJ and JL supervised JM’s doctorate as primary and secondary supervisors, respectively.

Indigenous methodologies require researchers to examine their relationship to their research (Kovach, 2021; Tuhiwai Smith, 2013). We (study team members who were not Quw'utsun) learned about Quw'utsun and other Indigenous peoples’ stories of maternity and child rearing in books, reports, and oral histories. We dedicated time to unlearning patterns that privilege systemic colonialism and white supremacy (Jongbloed et al., 2023). Several community-based Indigenous research frameworks taught us about relationality and the significance of using cultural- and context-specific research frameworks when working alongside Indigenous communities (Funnell et al., 2020; Mashford-Pringle et al., 2023; Ward et al., 2020).

### Knitting the snuw'uy'ulh with research principles

#### Stsi'elh stuhw tu Sul-hween (honour the Elders)

In Quw'utsun, Sul-hween are honoured as knowledge holders of snuw'uy'ulh received from their ancestors and share these ways of life with younger generations to guide them through life. LS and DP guided all study activities in the same way, through sharing snuw'uy'ulh with the team. Together, we determined the type and timing of our data collection. LS expressed that “a medical record does not tell the whole story”, and this led us to gather qualitative data through Quw'utsun people’s stories before reviewing medical records.

Following Quw'utsun protocol that invitations for work are done in person, LS and DP visited a prospective participant for one-to-one interviews at their home, where possible. LS and DP nurtured trust between prospective participants and interviewers who were not *Hwulmuhw* and unknown to study participants. Food was provided for all study activities because Sul-hween teach that, since time immemorial, Sul-hween offered food to travelers (who used to arrive by canoe) before work began. For sharing circles, the study team, staff at Ts'ewulhtun Health Centre, and community volunteers prepared a traditional seafood dinner (such as crab and oysters), and followed Quw'utsun protocol to serve Sul-hween first. Research discussions began after the food was enjoyed. Sul-hween had the first and last words at community gatherings, also a Quw'utsun protocol. Sul-hween teach that xpey' (cedar) holds important cultural significance (Ronson, 2022). To reflect this, xpey' was placed on tables during study activities. We applied our early learnings of the *Hul'q'umi'num'* language to speak words and phrases in honour of the Sul-hween who are revitalizing the language.

#### Mukw' tu shhwa'luqwa 'o' tth'ele's tu shhwuli (family is the heart of life)

LS and DP co-designed study questionnaires to query the role of families on the occurrence of preterm birth. Sul-hween and mulen' (fathers) shared their perspectives and experiences with preterm birth in sharing circles. The study team fostered an environment of respect, trust, accountability, joy, and engagement of family. Research activities were rescheduled when community events took higher priority (such as a ceremony, illness, or death of a loved one).

#### Tl'i' to' mukw' mustimuhw (each person is important)

LS and DP invited participants with diverse lived experiences to share their story. Several participants scheduled an interview, cancelled, and participated months later. We waited until the participant was available. To increase accessibility for community members, study activities took place in spaces that were familiar to participants and transportation was offered through Ts'ewulhtun Health Centre.

#### Ts'its'uw'atul. Nuts'umat kwun' syaays sh-hwuys kwtho' mukw' (help one another and work together for the good of all)

In keeping with principles of self-determination and reciprocity in Indigenous health research, the study team responded to community-identified needs and priorities arising from research participants. When study participants requested to learn more about snuw'uy'ulh, the Study team and Hwialusmutul' added a monthly “Tea with Sul-hween” to a regularly scheduled prenatal health promotion program to share snuw'uy'ulh with those pregnant or postpartum.

Two concurrent public health crises occurred in BC during the study period: the toxic drug crisis and the COVID-19 pandemic. To uphold community self-determination in health emergency response, the study team supported local health promotion activities for harm reduction in relation to substance use and vaccine clinics for COVID-19. Activities were paused during the COVID-19 pandemic beyond provincial public health restrictions to respect the community’s health and safety.

#### Thuthi'stuhw tun' shqwaluwun 'u tun' ts'lhmustimuhw (be true with your feelings and to another)

The study was based at Ts'ewulhtun Health Centre and funding was held and administered by Cowichan Tribes to assert the community’s leadership. To respect Quw'utsun right to control data, all primary data were collected and stored on Quw'utsun territory or accessed remotely through a secure network. Secondary analysis of administrative data was granted after review and approval from data stewards: FNHA and BC Ministry of Health.

### Knowledge translation

Indigenous scholars describe how research is “inextricably linked to action” (Smylie et al., 2014). We conducted several activities to respect the right of Quw'utsun mustimuhw to follow our progress and receive results in a timely manner. A logo created by a Quw'utsun artist strengthened recognition of the project on knowledge translation activities. Our progress was shared in weekly meetings with *Hwialusmutul'*, to all community members through the Quw'utsun Newsletter, and at community events.

The Research Advisory Committee reviewed knowledge translation materials before results were shared. For community review of results, we held a dinner and conversation with Sul-hween, presented to the Health Advisory Committee, and held a community dinner. We disseminated results with Island Health and FNHA in meetings, and to the public health community in presentations and at academic conferences.

To uphold snuw'uy'ulh, oral presentations were co-delivered by Sul-hween and other team members. Sul-hween opened and closed each presentation and spoke first on each component. Team members prepared visual slides and spoke to individual aspects of the study.

## Outcome: Ti'tul'atul' tst (learn from one another)

*Nuts'a'maat shqwaluwun* created a research environment where we could *Ti'tul'atul' tst* (learn from one another). This learning took time (5 years) and allowed us to bring our distinct knowledges and backgrounds together to collect and analyze data, and conduct knowledge translation activities, in ways that respected snuw'uy'ulh. As a result, *Nuts'a'maat shqwaluwun* advanced the community’s goal to “bring healthy birth back”.

For data collection, *Nuts'a'maat shqwaluwun* made it possible to conduct one-to-one interviews with 29 Quw'utsun *slhunlheni'* (women) who delivered term and preterm gestations to understand *tuletun'* (mothers) perspectives of birth. We also conducted two sharing circles with Sul-hween and *mulen'* (fathers), respectively, to understand their perspectives on preterm birth and pregnancy. Potential participants were willing to participate because LS and DP followed cultural protocol (through in-person invitations) and described the importance of the study. We conducted a population-based retrospective cohort study using a linkage of the First Nations Client File (FNCF) and BC Perinatal Data Registry to determine whether there were clinical or health service delivery issues documented in medical records that may be associated with preterm birth. The FNCF is a cohort of First Nations in BC registered under the Indian Act, and their unregistered descendants for whom entitlement-to-register can be determined, linkable to the provincial personal health number (First Nations Health Authority, 2020). This first-ever data access request was approved by co-stewards of the FNCF (FNHA and BC Ministry of Health) because the study was community-led and respected snuw'uy'ulh.

*Nuts'a'maat shqwaluwun* fostered strong partnerships with FNHA, Island Health, and UBC, and these partnerships enabled us to achieve our research and knowledge translation objectives. FNHA staff promoted action on preventing preterm birth among First Nations in BC and connected us to their related initiatives toward advancing perinatal health and wellness. Co-leadership from FNHA led to a first-ever report of preterm birth in the Cowichan Valley Region, disaggregated by First Nations status. Island Health team members invited us to present to local health providers and in a public seminar. JM received academic supervision and funding from UBC as a PhD student. All partners (FNHA, Island Health, and UBC) prepared policy recommendations for health system organizations based on our results.

Quw'utsun mustimuhw told us *naan 'o' tl'i' s-yaays* (this work is meaningful), in that Quw'utsun led the study and snuw'uy'ulh were respected. This feedback was received through community forums and personal conversations.

## Implications

We recognize that the inherent and legislated right of Indigenous Peoples to self-determination includes the right to lead research involving them (Smylie et al., 2020). In our experience, several principles of *Nuts'a'maat shqwaluwun* enabled Quw'utsun mustimuhw to lead this research.

1. *Nuts'a'maat shqwaluwun* meant honouring the Elders and working with open hearts to “learn from one another”. This way of working created trust within our team. It is important that public health practitioners foster trusting relationships with Indigenous communities, as described in TCPS-2 (Government of Canada, 2022).
2. Quw'utsun mustimuhw found the study to be meaningful because we followed snuw'uy'ulh. Learning Hul'q'umi'num' immeasurably strengthened our understanding of snuw'uy'ulh. We recommend that public health organizations employ cultural and language educators, ensure access to these resources, incorporate Indigenous languages into meetings, and follow guidance from health leaders to advance the rights of Indigenous people (Jongbloed et al., 2023).
3. This study revealed a lack of data to investigate the community’s concern about preterm birth. Few jurisdictions in Canada identify Indigenous people in routinely collected data. To determine their own health goals, Indigenous communities require data that are disaggregated by Indigenous identity, are jointly governed by Indigenous communities, reflect Indigenous ways of knowing, and are available in a timely manner (Hayward et al., 2021; Smylie et al., 2010).
4. Learning from one another created opportunities for community members to lead. However, team members who were trained in research methods were not Quw'utsun people. Colonialism severely impacts educational opportunities for Indigenous people (Government of Canada, 2015). We recommend that health system organizations partner with Indigenous-led research mentorship programs (Erb et al., 2024) to support the next generation of Indigenous health researchers.

## Implications for policy and practice

What are the innovations in this policy or program?

- Our framework of working together as *nuts'a'maat shqwaluwun* (one heart, one mind) enabled a partnership between a First Nations community, public health organizations, and a university to learn from one another. This framework supported the community’s research goal to “bring healthy birth back to Quw'utsun” and consequently supported the inherent right of Quw'utsun people to determine their own research priorities and lead research about their community.
- Following Quw'utsun ways of life led to research that was meaningful to the community.

What are the burning research questions for this innovation?

- How can partnerships between public health and Indigenous communities support Indigenous communities to define and reach their health and wellness goals?
- How can public health ensure that routinely collected data are disaggregated by Indigenous identity to enable Indigenous communities to define their own health and wellness? How can public health data include indicators that matter to Indigenous communities and reflect Indigenous ways of knowing?

## Data Availability

Not applicable.

## Footnotes

1 Quw'utsun Elder T'ultunaat (Lydia Seymour) died in August 2024.
